# Clinical electrophysiology of vision—commentary on current status and future prospects

**DOI:** 10.1038/s41433-021-01592-0

**Published:** 2021-05-27

**Authors:** Ruth Hamilton

**Affiliations:** 1 Department of Clinical Physics and Bioengineering, NHS Greater Glasgow & Clyde, Glasgow, UK; 2 College of Medical, Veterinary and Life Sciences, University of Glasgow, Glasgow, UK

**Keywords:** Visual system, Diagnosis

Visual electrophysiology is a powerful, non-invasive tool in ophthalmology and neurology. It is of paramount importance in the diagnosis of diseases affecting the visual pathway because it objectively and specifically assesses function, localising dysfunction to the retinal pigment epithelium, retina, macula, optic nerve, chiasm or higher visual processing centres [1]. Crucially, it bridges objective structural information provided by imaging and examination, and psychophysical clinical observations such as acuity or visual fields. Clinical visual electrophysiology, therefore, is a necessary component of ophthalmic and neurological practice.

Outwith the clinic, visual electrophysiology delivers objective and quantifiable data and therefore is increasingly used in deep phenotyping and in clinical trials: at the time of writing, over 200 active clinical trials are using electrophysiological outcome measures. It is particularly helpful in animal models and hence has been instrumental in delivering some of the key therapies for sight-threatening disease [2].

By 1958, clinical electroretinography had reached the critical mass needed to form a society, the International Society for Clinical Electroretinography (ISCERG). At its first meeting in 1961, it was recognised that “the most urgent task of the Society is to establish certain standards” [3]. Ragnar Arthur Granit, a founding member of ISCERG, shared the 1967 Nobel Prize in Physiology or Medicine for using the electroretinogram (ERG) to discover “primary physiological and chemical visual processes in the eye”. In his Nobel Lecture, he praised Gösta Karpe, another ISERG founder, for making the ERG a “useful clinical method, nowadays considerably developed and employed all over the world” [4]. These early descriptions of clinical methods paved the way for an international standard [5–7]. The critical role of the ERG for understanding retinitis pigmentosa led to a collaboration between the Society, by then named the International Society for Clinical Electrophysiology of Vision (ISCEV), and the National Retinitis Pigmentosa Foundation of the USA. Led by Michael F. Marmor, the first international ERG Standard [8] was published in 1989.

The ERG Standard [9] is being revised for the seventh time, and four further Standards, for the visual evoked potential (VEP) [10], the pattern ERG [11], the multifocal ERG [12] and the electrooculogram [13], are updated periodically by ISCEV. Recognising that these Standards each describe a minimum test set, and in response to an international survey of actual practice, eight extended protocols have also been published. These extend standard testing to specific cortical or retinal functions; for example, the VEP can be used to estimate visual acuity [14]. On- and off-pathway testing aids in the diagnosis of congenital stationary night-blindness, melanoma-associated retinopathy, phosphomannomutase deficiency and autoimmune retinopathy [15]. S-cone ERGs can refine diagnosis in enhanced S-cone syndrome, distinguish rod monochromacy from S-cone monochromacy, and may help diagnose inherited tritanopia or elevated tritan thresholds [16]. Stimulus-response series of light- or dark-adapted ERGs add information about the aetiology or prognosis of diseases primarily affecting cone [17] or rod system function [18]. Dark-adapted red flash ERGs aid diagnosis of rod- and S-cone monochromacy, cone dystrophy, vitamin A deficiency, RDH5-retinopathy, SAG- or GRK1-retinopathy, some cases of rod-cone dystrophy, RGS9- and R9AP-retinopathy and colour vision deficiencies [19]. The photopic negative response reveals ganglion cell pathology in patients with glaucoma, optic atrophy, central retinal artery occlusion, ischaemic optic neuropathy, diabetic retinopathy, and idiopathic intracranial hypertension [20]. The strong flash rod-isolated ERG a-wave can aid understanding of diseases affecting the rod system: this extended protocol is ready to facilitate the move from research to clinic [21].

The primary aim of standards in clinical visual electrophysiology was to ensure that recordings between clinics or centres were comparable [3], and the evidence suggests this aim has been met: a multi-centre ERG study conducted in the UK demonstrated remarkably low variability, corresponding with acceptable variability of biochemical assays [22]. The Standards represent a cornerstone of clinical visual electrophysiology, embedding comparability and repeatability in the tests to an extent rarely achieved in clinical or laboratory measurement.

What lies ahead? Healthcare is changing, albeit differently in different parts of the world. What should clinical electrophysiology look like as we move away from episodic, illness-driven healthcare encounters, towards universal genotyping for susceptibility to ophthalmic disease? What should it look like in developing economies or in remote communities? What should it look like in a data-driven world, and an imaging-driven world? The service delivery model will need to diversify: retaining complex, detailed assessments at specialist centres, while developing alternative, accessible ways to deliver these essential tests to the huge numbers of people who cannot yet access their benefit. The tests need to be quicker and easier, diagnostically more robust, less onerous for patients and trial subjects, and more widely available. Change is already happening, as handheld and more compact systems arrive on the market, bringing clinical visual electrophysiology into theatres, wards, intensive care settings and even community settings. Innovations transforming other areas of clinical measurement—wireless electrodes [23], virtual reality, advanced signal processing [24], eye-tracking, machine learning, harmonised reference data [25]—have yet to impact clinical visual electrophysiology. The opportunity exists to leverage the diverse scientific, technical, clinical and commercial skills of the visual electrophysiology community and evolve these important tests for 21st century healthcare.
